# Label-free luminescence switch-on detection of hepatitis C virus NS3 helicase activity using a G-quadruplex-selective probe†
†Electronic supplementary information (ESI) available: Compound characterisation and supplementary data. See DOI: 10.1039/c4sc03319a



**DOI:** 10.1039/c4sc03319a

**Published:** 2014-11-25

**Authors:** Ka-Ho Leung, Hong-Zhang He, Bingyong He, Hai-Jing Zhong, Sheng Lin, Yi-Tao Wang, Dik-Lung Ma, Chung-Hang Leung

**Affiliations:** a Department of Chemistry , Hong Kong Baptist University , Kowloon Tong , Hong Kong , China . Email: edmondma@hkbu.edu.hk; b State Key Laboratory of Quality Research in Chinese Medicine , Institute of Chinese Medical Sciences , University of Macau , Macao , China . Email: duncanleung@umac.mo

## Abstract

A series of luminescent Ir(iii) complexes were synthesised and evaluated for their ability to act as luminescent G-quadruplex-selective probes. The Ir(iii) complex **9**, [Ir(phq)_2_(phen)]PF_6_ (where phq = 2-phenylquinoline; phen = 1,10-phenanthroline), exhibited high luminescence in the presence of G-quadruplex DNA compared to dsDNA and ssDNA, and was employed to construct a label-free G-quadruplex-based assay for hepatitis C virus NS3 helicase activity in aqueous solution. Moreover, the application of the assay for screening potential helicase inhibitors was demonstrated. To our knowledge, this is the first G-quadruplex-based assay for helicase activity.

## Abstract 2

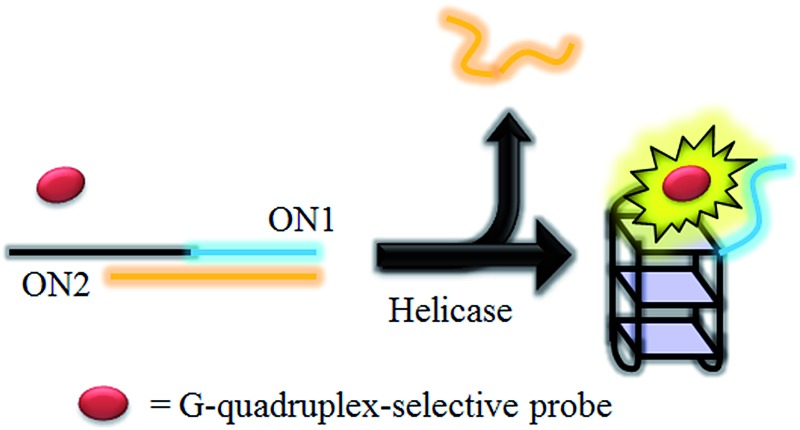
A novel luminescent G-quadruplex-selective iridium(iii) complex was employed in a label-free G-quadruplex-based detection assay for hepatitis C virus NS3 helicase activity.

## Introduction

Helicases unwind dsDNA and dsRNA, and displace nucleic acid-binding proteins by using energy from ATP hydrolysis.^1,2^ Helicase is an essential enzyme in cells for the reading, replication, and repair of genomes. However, helicases are also implicated in a number of viral diseases due to their critical role in facilitating viral replication and proliferation.^3^ Viral helicase inhibitors have been developed for the treatment of hepatitis C and herpes simplex viral infections.^4^ Due to its biological and medical importance, the development of efficient assays for monitoring the nucleic acid unwinding activity of helicase is of great interest.

Conventional techniques for the detection of helicase activity typically involve radioactive labeling in conjunction with gel electrophoresis.^5–7^ However, this method is discontinuous, time-consuming, inefficient, and necessitates the use of stringent safety procedures to control radiographic exposure, thus limiting the scope of its application. Meanwhile, rapid advances in the field of DNA technology over the past several years have highlighted the potential use of oligonucleotides as attractive signal transducing units for the detection of biologically and environmentally important analytes.^8–17^ Oligonucleotides offer salient advantages in biosensing applications, such as their relatively small size, low cost, facile synthesis and modification, good thermal stability, and reusability.^18–26^ The G-quadruplex motif, which is a non-canonical DNA secondary structure composed of planar stacks of four guanines stabilized by Hoogsteen hydrogen bonding,^27–30^ has attracted particular interest in sensing applications.^31,32^ The extensive structural polymorphism of G-quadruplexes has rendered them versatile signal-transducing elements for the development of DNA-based probes.^33–48^

In recent years, a number of luminescent oligonucleotide-based sensing platforms for helicase activity have been developed.^49–53^ For example, Min and co-workers reported a fluorescent assay for hepatitis C virus (HCV) NS3 helicase activity and severe acute respiratory syndrome coronavirus (SARS-CoV, SCV) helicase nsP13 activity by utilizing graphene and a fluorescently labeled dsDNA substrate.^54,55^ Ali and co-workers used SYBR Green dye, which is strongly fluorescent in the presence of dsDNA but not ssDNA, for the detection of helicase activity.^56^ A similar principle was utilized by Kowalczykowski and co-workers to construct a switch-off platform for helicase activity using ethidium bromide, ethidium homodimer, bis-benzimide (DAPI), Hoechst 33258 or thiazole orange. The groups of Frick and Boguszewska-Chachulska reported an approach for monitoring helicase activity using molecular beacons.^57,58^ Recently, Balci and co-workers used single-molecule Forster resonance energy transfer (FRET) imaging to monitor helicase activity.^59–61^ These reports demonstrate that DNA oligonucleotides can be integrated as useful functional and structural elements for the construction of sensitive “switch-on” luminescent platforms for the detection of helicase.

**Fig. 1:**
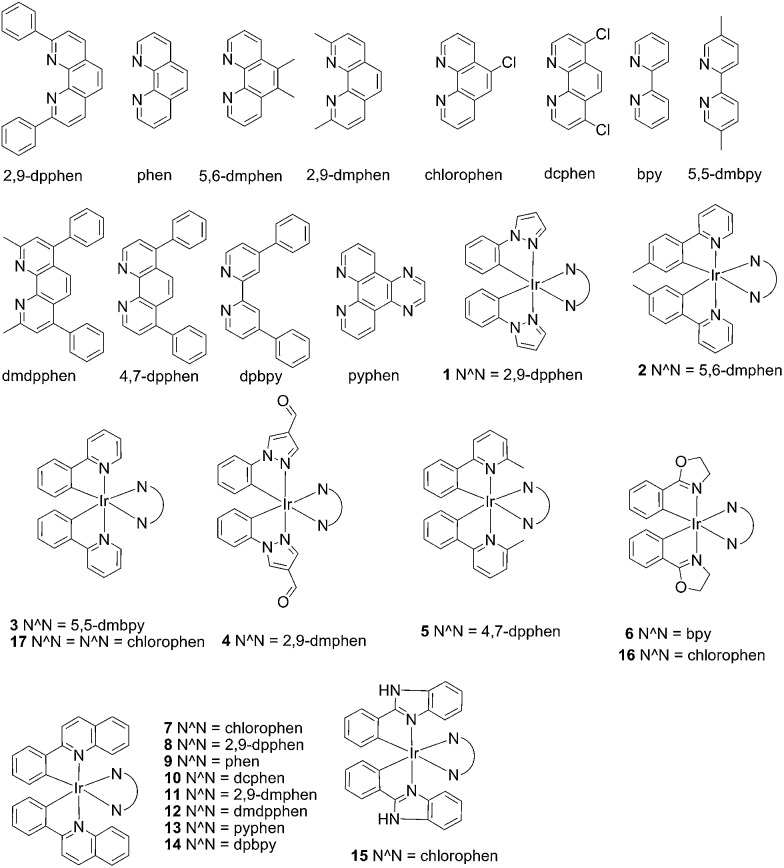
Chemical structures of the luminescent Ir(iii) complexes **1–17** that were synthesised and evaluated in this study.

In recent years, luminescent transition metal complexes have arisen as viable alternatives to organic dyes for sensory applications due to their notable advantages.^62–70^ Firstly, metal complexes generally emit in the visible region with a long phosphorescence lifetime, allowing them to be readily distinguished from a fluorescent background arising from endogenous fluorophores in the sample matrix by the use of time-resolved fluorescence spectroscopy. Secondly, the precise and versatile arrangement of co-ligands on the metal centre allows the interactions of metal complexes with biomolecules to be fine-tuned for maximum selectivity and sensitivity. Thirdly, these metal complexes often possess interesting photophysical properties that are strongly affected by subtle changes in their local environment. For example, Pt(ii)^71–73^ and Ru(ii)^74–77^ complexes have been extensively investigated as “molecular light switches” for nucleic acids, including G-quadruplex DNA. However, luminescent complexes based on the Ir(iii) center have been comparatively less explored. In this study, a series of luminescent Ir(iii) complexes were synthesised and evaluated for their ability to act as G-quadruplex-selective luminescence switch-on probes. The iridium(iii) complex **9**, [Ir(phq)_2_(phen)]PF_6_ (where phq = 2-phenylquinoline; phen = 1,10-phenanthroline), (Fig. 1) was employed as a G-quadruplex probe for the construction of a label-free luminescent detection platform for helicase activity in aqueous solution, utilizing hepatitis C virus NS3 helicase as a model enzyme. To our knowledge, no luminescent G-quadruplex-based assay for the detection of helicase activity has yet been reported in the literature.

## Results and discussion

### Principle of the luminescent G-quadruplex-based probe for HCV NS3 helicase activity

**Scheme 1:**
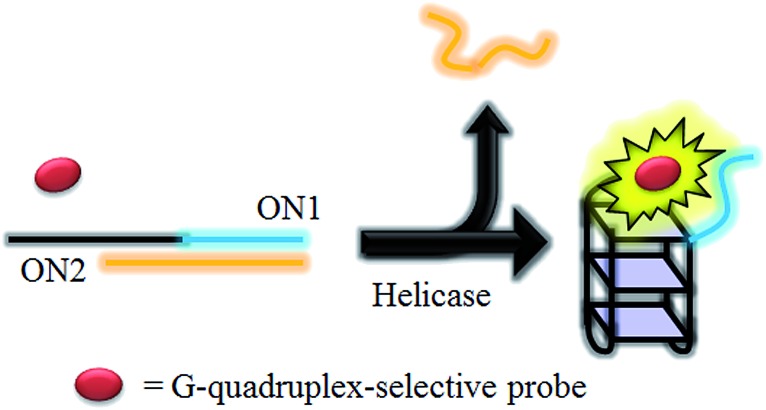
Schematic diagram of the luminescent switch-on assay to monitor the duplex-DNA unwinding activity of helicase using a G-quadruplex-selective probe.

HCV NS3 helicase is able to unwind dsDNA into ssDNA, and is one of the essential enzymes required for the replication of HCV.^78^ The proposed mechanism of the HCV NS3 helicase activity assay is outlined in Scheme 1. We designed a double-stranded oligomer consisting of a G-quadruplex-forming sequence (ON1, 5′-GTG_3_TAG_3_CG_3_T_2_G_2_TG_2_CGACG_2_CAGCGAG_2_CAGAG_2_AGCAGAG_3_AGCA-3′) and its complementary cytosine-rich sequence (ON2, 5′-GC_2_TCGCTGC_2_GTCGC_2_AC_2_A_2_C_3_GC_3_-3′), which acts as a substrate for helicase. In the absence of helicase, the double-stranded oligonucleotide substrate is not unwound, and remains as a duplex structure that interacts only weakly with the luminescent Ir(iii) complex. However, in the presence of helicase, unwinding of the duplex DNA substrate by helicase generates two ssDNA fragments. After the reaction is stopped by the addition of EDTA,^51^ the G-quadruplex-forming oligomer ON1 is able to fold into a G-quadruplex motif in the presence of K^+^ ions. The nascent G-quadruplex structure is then recognized by the luminescent Ir(iii) complex with an enhanced emission response, allowing the system to function as a switch-on luminescent probe for helicase activity.

### Screening of iridium(iii) complexes as G-quadruplex-selective probes

**Fig. 2:**
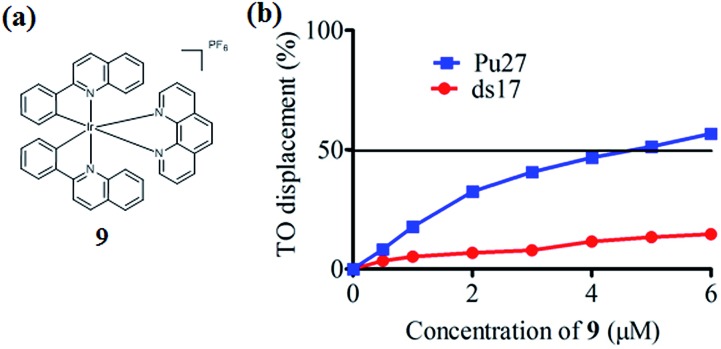
(a) Chemical structure of complex **9**; (b) G4-FID titration curves of DNA duplex ds17 and Pu27 G-quadruplex in the presence of increasing concentrations of complex **9** in Tris–HCl buffer. The DC_50_ value is equal to the half-maximal concentration of compound required to displace 50% TO from DNA.

In the present study, a library of seven luminescent Ir(iii) complexes (**1–7**, Fig. 1) were initially examined for their emission response to different forms of DNA, including G-quadruplex, ssDNA and dsDNA (Table S1†). Of these seven complexes, only complex **7** bearing the N^N ligand chlorophen (5-chloro-1,10-phenanthroline) and the C^N ligand phq showed a selective response for G-quadruplex DNA (Fig. S1†), while not showing any luminescence enhancement in the presence of helicase (data not shown). On the other hand, complexes **3**, **5**, and **6** were found to be non-selective for G-quadruplex DNA (Fig. S1†), whereas the luminescence of **1**, **2**, and **4** was enhanced in the presence of helicase only (data not shown). Based on the structure of complex **7**, a focused library of eleven Ir(iii) complexes (**7–17**, Fig. 1) containing phq and chlorophen derivatives as ligands were designed and synthesised. Encouragingly, the second round of screening revealed that the Ir(iii) complex **9** displayed a significantly enhanced luminescence response in the presence of the PS2.M G-quadruplex (Fig. S2†), and no luminescence enhancement in the presence of helicase (Fig. S4†). Meanwhile, complexes **14–17** were non-selective for G-quadruplex DNA (Fig. S1†), while the luminescence of **8**, and **10–13** was enhanced on addition of helicase only (data not shown). To further validate the suitability of **9** as a G-quadruplex-selective probe, we performed G-quadruplex fluorescent intercalator displacement (G4-FID) and fluorescence resonance energy transfer (FRET) melting assays to determine the selectivity of **9** for G-quadruplex DNA. The G4-FID assay showed that complex **9** was able to displace thiazole orange (TO) from G-quadruplex DNA (^G4^DC_50_ = *ca.* 5 μM, half-maximal concentration of compound required to displace 50% TO from DNA) with higher efficacy than from duplex DNA (Fig. 2b). Additionally, FRET melting assays revealed that the melting temperature (Δ*T*_m_) of the F21T G-quadruplex was increased by about 13 °C upon the addition of complex **9** (Fig. S3a†). In comparison, only a 4 °C change in the melting temperature of F10T dsDNA was observed for the same concentration of complex **9** (Fig. S3†). Taken together, these results demonstrate the ability of complex **9** to discriminate between G-quadruplex DNA and dsDNA or ssDNA. The luminescence enhancement of complex **9** in the presence of G-quadruplex DNA is presumably due to its ability to bind to G-quadruplex structures through groove/loop binding or end-stacking interactions. This shields the complex from the aqueous solvent environment and suppresses non-radiative decay of the excited state, thus leading to enhanced triplet state emission.

### Luminescent detection of HCV NS3 helicase activity in aqueous solution

The characterization and photophysical properties of the Ir(iii) complexes **1–17** are given in the ESI (Table S2†). Given the promising G-quadruplex-selective luminescence behaviour exhibited by complex **9**, we sought to employ **9** as a G-quadruplex-selective probe to construct a label-free luminescent detection platform for helicase activity in aqueous solution. We first investigated the luminescence response of complex **9** and the ON1-ON2 duplex substrate to helicase. Upon incubation with helicase, the luminescence of **9** was significantly enhanced. We hypothesize that the luminescence enhancement of the system was due to the unwinding of the duplex DNA substrate by helicase, which allowed the formation in the presence of K^+^ of the G-quadruplex motif that was subsequently recognized by **9**. To verify the mechanism of the proposed assay, a number of control experiments were conducted. We first incubated complex **9** with helicase in the absence of the duplex DNA substrate. No luminescence enhancement was observed, indicating that complex **9** did not directly interact with helicase (Fig. S4†). We also designed mutant DNA sequences (ON1_m_, 5′-GT*ATA*TA*TAC*CG_3_T_2_G_2_TG_2_CGACG_2_CAGCGAG_2_CAGAG_2_AGCAGAG_3_AGCA-3′) that are unable to form a G-quadruplex structure in the presence of helicase, due to the lack of key guanine residues. We observed a slight decrease in the luminescence of **9** in response to helicase in the presence of the mutant DNA sequences, indicating that the formation of the G-quadruplex motif was important for the luminescence enhancement of the system (Fig. S5†). Taken together, these results suggest that the luminescence enhancement of the system originated from the specific interaction of **9** with the G-quadruplex motif, which was generated by the unwinding of the duplex DNA substrate by helicase.

Various studies have been performed to investigate the ability of helicases to unfold G-quadruplex structures. One study reported that Bloom's syndrome helicase (BLM) could unfold telomeric G-quadruplex in the absence of ATP,^60^ while another study reported that BLM translocation was hindered by G-quadruplex motifs, with the unwinding efficiency being dependent on the stability of the G-quadruplex structure, which was in turn influenced by loop size or ionic strength.^79^ For example, the unfolding activity of BLM towards a particular G-quadruplex sequence was completely stopped in 150 mM K^+^.^60^ Therefore, it was important to investigate whether HCV NS3 helicase could unfold the G-quadruplex structure used in this study. The results showed that no significant change in the luminescence intensity of the **9**/G-quadruplex ensemble was observed upon the addition of 0.8 μM HCV NS3 helicase, indicating that this helicase did not unfold the G-quadruplex structure employed in this study (Fig. S6†). However, further investigation and optimization may be required for the detection of other helicases.

**Fig. 3:**
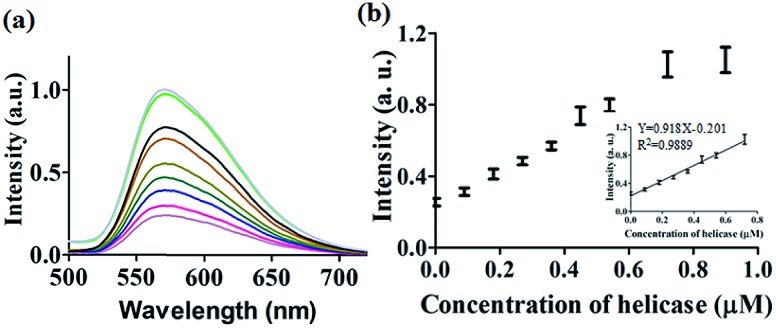
(a) Luminescence spectra of the **9**/G4-quadruplex system in response to increasing concentrations of helicase: 0, 0.09, 0.18, 0.27, 0.36, 0.45, 0.54, 0.72, and 0.9 μM. (b) The relationship between luminescence intensity at *λ* = 571 nm and helicase concentration. Inset: linear plot of the change in luminescence intensity at *λ* = 571 nm *vs.* helicase concentration.

After optimization of the concentrations of complex **9** (Fig. S7†), DNA (Fig. S8†) and ATP (Fig. S9†), we investigated the luminescence response of the system to different concentrations of helicase. The system exhibited a *ca.* 4.5-fold enhancement in luminescence at [helicase] = 0.9 μM (Fig. 3a), with a linear range of helicase detection from 0 to 0.72 μM (Fig. 3b). Furthermore, the detection limit of this assay for helicase was estimated to be 0.09 μM with a signal-to-noise ratio (*S*/*N*) of 3 (Fig. S10†).

### Selectivity of the G-quadruplex-based HCV NS3 helicase activity assay

**Fig. 4:**
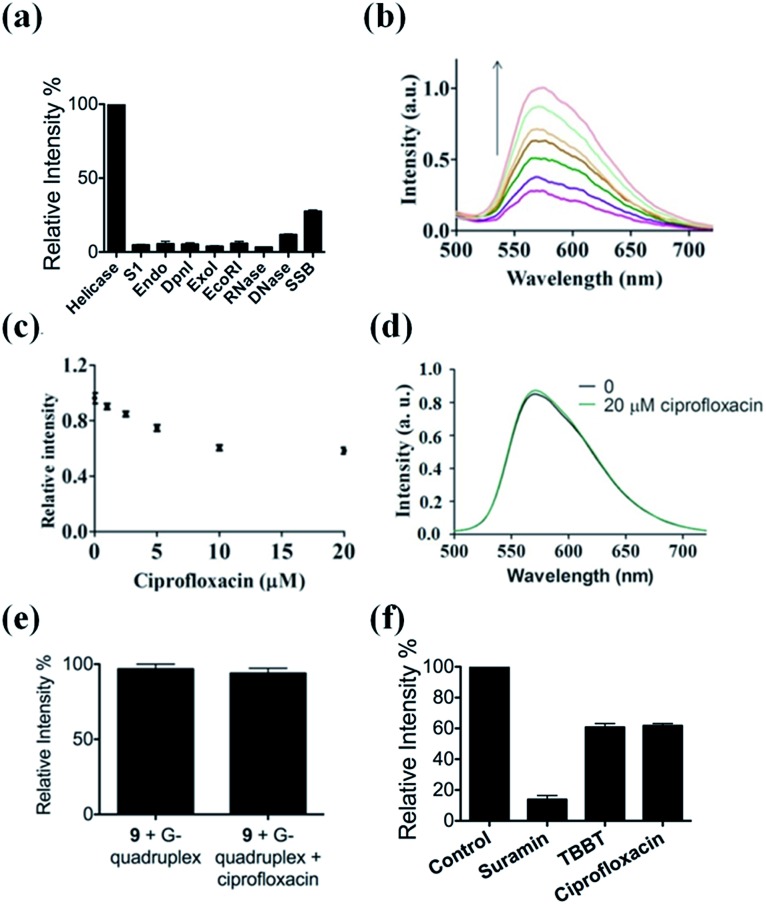
(a) Luminescence response of the system in the presence of helicase, S1, Endo, DpnI, ExoI, EcoRI, RNase, DNase and SSB. (b) Luminescence spectra of the **9**/G-quadruplex system in a reaction system containing 0.5% (v/v) cell extract in response to various concentrations of helicase: 0, 0.18, 0.36, 0.45, 0.54, 0.72, and 0.9 μM. (c) Relative luminescence intensities of the system in the presence of increasing concentrations of ciprofloxacin: 0, 1, 2.5, 5, 10, and 20 μM. (d) Emission spectra of complex **9** in the absence and presence of 20 μM ciprofloxacin. (e) Relative luminescence response of the **9**/G-quadruplex ensemble upon the addition of 20 μM ciprofloxacin. (f) Luminescence response of the system treated with 0.8 μM helicase and 10 μM of suramin, TBBT and ciprofloxacin.

The selectivity of our approach to an HCV NS3 helicase activity assay was evaluated by investigating the response of the system to S1 nuclease (S1), endonuclease IV (Endo), DpnI, exonuclease I (ExoI), EcoRI, RNase, DNase and single-stranded DNA binding protein (SSB). The results showed that only helicase could significantly enhance the luminescence of the complex **9**/G-quadruplex DNA system (Fig. 4a). No significant change in emission intensity was observed upon addition of the nucleases, while a relatively low emission enhancement was observed for single-stranded DNA binding protein. These results indicate that the system displays selectivity for helicase over nucleases or single-stranded DNA binding proteins, which presumably originates from the unwinding of the duplex DNA substrate by helicase.

### Application of the HCV NS3 helicase activity detection assay in biological samples

To evaluate the robustness of the system, we investigated the performance of our G-quadruplex-based sensing platform for helicase activity in the presence of cellular debris. In a reaction system containing 0.5% (v/v) cell extract, the **9**/G-quadruplex DNA system exhibited a gradual increase in luminescence intensity as the concentration of helicase increased (Fig. 4b). This result demonstrates that this assay could potentially be further developed for the detection of helicase unwinding activity in biological samples.

### Application of the HCV NS3 helicase activity detection assay in inhibitor screening

The utility of this G-quadruplex-based assay for screening potential helicase inhibitors was also studied. Here, ciprofloxacin was chosen as a model inhibitor of helicase.^56^ The luminescence signal of the system was diminished in the presence of ciprofloxacin in a dose-dependent manner, with a decrease of about 40% observed at a ciprofloxacin concentration of 10 μM (Fig. 4c). The inhibitory effect of ciprofloxacin on helicase activity in our assay is comparable to that reported by Ali and co-workers.^56^ Furthermore, ciprofloxacin has no direct quenching effect on the luminescence of **9** (Fig. 4d) or the **9**/G-quadruplex ensemble (Fig. 4e). To further demonstrate the application of this platform for inhibitor screening, we investigated a group of well-known HCV NS3 helicase inhibitors.^80^ The inhibitors tested displayed inhibitory activity towards HCV NS3 helicase in this platform, without having a direct quenching effect on **9** or the **9**/G-quadruplex ensemble (Fig. 4f and S11†). These results further validate our detection platform as a screening tool for HCV NS3 helicase inhibitors.

## Conclusions

In conclusion, a library of 17 luminescent Ir(iii) complexes containing various C^N and N^N ligands were screened for their ability to act as G-quadruplex probes. Ir(iii) complex **9** was discovered to be a G-quadruplex-selective luminescent probe, and a label-free luminescent assay for helicase activity was developed utilizing the G-quadruplex-selective property of **9**. Compared to previously reported radiographic or luminescent assays that require multiple steps and/or the use of isotopically or fluorescently labeled nucleic acids, our label-free approach is less time consuming and more cost-effective, as expensive and tedious pre-labeling or immobilization steps are avoided. On the other hand, the labeling of an oligonucleotide with a fluorophore may disrupt the interaction between the oligonucleotide and its cognate target. Finally, our developed label-free DNA-based detection platform employs luminescent transition metal complexes, which offer several advantages compared to the relatively more popular organic fluorophores, such as long phosphorescence lifetimes, large Stokes shift values and modular syntheses. Additionally, the assay could function effectively in diluted cell extract, and its potential application in the screening of helicase inhibitors was also demonstrated, though further optimisation may be required. We envision that our novel switch-on, label-free G-quadruplex-based luminescent detection method for helicase activity could potentially be developed as a useful tool in biochemical and biomedical research.
