# Supplementary material for: Label-free luminescence switch-on detection of hepatitis C virus NS3 helicase activity using a G-quadruplex-selective probe
Source: Chem Sci. 2014 Nov 25;6(4):2166–71. doi: 10.1039/c4sc03319a (PMC5539802; doi:10.1039/c4sc03319a)
Supplement: Supplementary file 1 [file SC-006-C4SC03319A-s001.pdf]

Electronic Supplementary Material (ESI) for Chemical Science.
This journal is © The Royal Society of Chemistry 2015

Electronic Supporting Information

Label-free luminescence switch-on detection of hepatitis C virus NS3 helicase activity using a G-quadruplex-selective probe

Ka-Ho Leung,‡[a] Hong-Zhang He,‡[a] Bingyong He,[a] Hai-Jing Zhong,[b] Sheng Lin,[a] Yi-Tao Wang,[b] Dik-Lung Ma*[a] and Chung-Hang Leung*[b]

[a] *Department of Chemistry, Hong Kong Baptist University, Kowloon Tong, Hong Kong, China. E-mail: edmondma@hkbu.edu.hk*

[b] *State Key Laboratory of Quality Research in Chinese Medicine, Institute of Chinese Medical Sciences, University of Macau, Macao, China. E-mail: duncanleung@umac.mo*

Experimental section

Materials

Reagents, unless specified, were purchased from Sigma Aldrich (St. Louis, MO) and used as received. Iridium chloride hydrate ($IrCl_3.xH_2O$) was purchased from Precious Metals Online (Australia). Recombinant HCV NS3 helicase was expressed and purified using p24a-NS3 H plasmid. HCV cDNA encoding the NS3 helicase protein (amino acids 1193 to 1659 of the polyprotein encoded by genotype 1b) was inserted in the multiple cloning site of vector pET24a (Novagen). S1 nuclease (S1), endonuclease IV (Endo), DpnI, exonuclease I (ExoI), EcoRI, RNase, DNase, single-stranded DNA binding protein (SSB) was purchased from New England Biolabs Inc. (Beverly, MA, USA). All oligonucleotides were synthesized by Techdragon Inc. (Hong Kong, China).

General experimental

Mass spectrometry was performed at the Mass Spectroscopy Unit at the Department of Chemistry, Hong Kong Baptist University, Hong Kong (China). Deuterated solvents for NMR purposes were obtained from Armar and used as received.

^1H and ^{13}C NMR were recorded on a Bruker Avance 400 spectrometer operating at 400 MHz (^1H) and 100 MHz (^{13}C). ^1H and ^{13}C chemical shifts were referenced internally to solvent shift (acetone-d_6: ^1H δ 2.05, ^{13}C δ 29.8; CD_3Cl: ^1H δ 7.26, ^{13}C δ 76.8). Chemical shifts (δ) are quoted in ppm, the downfield direction being defined as positive. Uncertainties in chemical shifts are typically ±0.01 ppm for ^1H and ±0.05 for ^{13}C. Coupling constants are typically ± 0.1 Hz for ^1H-^1H and ±0.5 Hz for ^1H-^{13}C couplings. The following abbreviations are used for convenience in reporting the multiplicity of NMR resonances: s, singlet; d, doublet; t, triplet; q, quartet; m, multiplet; br, broad. All NMR data was acquired and processed using standard Bruker software (Topspin).

Photophysical measurement

Emission spectra and lifetime measurements for complexes were performed on a PTI TimeMaster C720 Spectrometer (Nitrogen laser: pulse output 337 nm) fitted with a 380 nm filter. Error limits were estimated: λ (±1 nm); τ (±10%); φ (±10%). All solvents used for the lifetime measurements were degassed using three cycles of freeze-vac-thaw.

Luminescence quantum yields were determined using the method of Demas and Crosby[1] $[Ru(bpy)_3][PF_6]_2$ in degassed acetonitrile as a standard reference solution (Φ_r = 0.062) and calculated according to the following equation:

$$\Phi_s = \Phi_r(B_r/B_s)(n_s/n_r)^2(D_s/D_r)$$

where the subscripts s and r refer to sample and reference standard solution respectively, n is the refractive index of the solvents, D is the integrated intensity, and Φ is the luminescence quantum yield. The quantity B was calculated by $B = 1 - 10^{-AL}$, where A is the absorbance at the excitation wavelength and L is the optical path length.

G4-FID assay

The FID assay was performed as previously described.[2] The Pu27 G-quadruplex DNA (0.25 μM) in Tris-HCl buffer (20 mM Tris, 100 mM KCl, pH 7.0) were annealed by heating at 95 °C for 10 min. Indicated concentration of thiazole orange (0.5 μM for

Pu27 G-quadruplex DNA and 0.5 μM for ds17) was added and the mixture was incubated for 1 h. Emission measurement was taken after addition of each indicated concentration of **9** followed by an equilibration time for 5 min. The fluorescence area was converted into percentage of displacement (PD) by using the following equation. $PD = 100 - [(FA/FA_0) \times 100]$ (FA_0 = fluorescence area of DNA-TO complex in the absence of **9**; FA = fluorescence area in the presence of **9**).

FRET melting assay

The ability of **9** to stabilize G-quadruplex DNA was investigated using a fluorescence resonance energy transfer (FRET) melting assay. The labelled G-quadruplex-forming oligonucleotide F21T (5′-*FAM*-d($G_3[T_2AG_3]_3$)-*TAMRA*-3′; donor fluorophore *FAM*: 6-carboxyfluorescein; acceptor fluorophore *TAMRA*: 6- carboxytetramethylrhodamine) was diluted to 200 nM in a potassium cacodylate buffer (100 mM KCl, pH 7.0), and then heated to 95 °C in the presence of the indicated concentrations of **9**. The labeled duplex-forming oligonucleotide F10T (5′-FAM-dTATAGCTA-HEG-TATAGCTATAT-TAMRA-3′) (HEG linker: $[(-CH_2-CH_2-O-)_6]$) was treated in the same manner, except that the buffer was changed to 10 mM lithium cacodylate (pH 7.4). Fluorescence readings were taken at intervals of 0.5 °C over the range of 25 to 95 °C.

Synthesis

The following complexes were prepared according to (modified) literature methods. All complexes are characterized by ^{1}H NMR, ^{13}C NMR, high resolution mass spectrometry (HRMS) and elemental analysis.

The precursor iridium(III) complex dimer $[Ir_2(C^\wedge N)_4Cl_2]$ is prepared as reported method[3]. Then, a suspension of $[Ir_2(C^\wedge N)_4Cl_2]$ (0.2 mmol) and corresponding N^N ligands 1,10-phenanthroline (phen), 2,9-diphenyl-1,10-phenanthroline (2,9-dpphen), 2,9-dimethyl-4,7-diphenyl-1,10-phenanthroline (dmdpphen), 5,6-dimethyl-1,10-phenanthroline (dmphen), 5-chloro-1,10-phenanthroline (chlorophen), or 4,7-dichloro-1,10-phenanthroline (dcphen), 2,2'-bipyridine (bpy), 5,5'-dimethyl-2,2'-bipyridine (5,5-dmbpy), 4,7-diphenyl-1,10-phenanthroline (4,7-dpphen), 4,4'-diphenyl-2,2'-bipyridine (dpbpy), pyrazino[2,3-f][1,10]phenanthroline (pyphen) (0.44

mmol) in a mixture of DCM:methanol (1:1, 20 mL) was refluxed overnight under a nitrogen atmosphere. The resulting solution was then allowed to cool to room temperature, and filtered to remove unreacted cyclometallated dimer. To the filtrate, an aqueous solution of ammonium hexafluorophosphate (excess) was added and the filtrate was reduced in volume by rotary evaoration until precipitation of the crude product occurred. The precipiate was then filtered and washed with several portions of water (2 × 50 mL) followed by diethyl ether (2 × 50 mL). The product was recrystallized by acetonitrile:diethyl ether vapor diffusion to yield the titled compound.

Complex **1**. Yield: 59%. ^{1}H NMR (400 MHz, Acetone-d_6) δ 8.11-8.09 (d, *J* = 8.0 Hz, 2H), 7.65-7.61 (m, 4H), 7.05-7.01 (d, *J* = 8.0 Hz, 2H), 6.49 (s, 2H), 6.36-6.32 (m, 2H), 6.15-6.03 (m, 10H), 5.86 (s, 2H), 5.69-5.67 (t, *J* = 8.0 Hz, 2H), 5.32-5.30 (t, *J* = 8.0 Hz, 2H), 4.33 (s, 2H); ^{13}C NMR (100 MHz, Acetone-d_6) δ 166.9, 150.1, 142.5, 140.6, 140.5, 139.6, 133.0, 131.3, 129.5, 129.3, 128.7, 128.6, 128.5, 128.1, 126.4, 121.9, 112.0, 108.6; HRMS: Calcd. for $C_{42}H_{30}IrN_6[M–PF_6]^+$: 811.2161 Found: 811.2142; Anal. ($C_{42}H_{30}N_6IrPF_6$) C, H, N: calcd 52.77, 3.16, 8.79; found 52.54, 3.20, 8.56.

Complex **2**. Reported[4]

Complex **3**. Yield: 53%. ^{1}H NMR (400 MHz, Acetone-d_6) δ 9.84 (d, *J* = 2.6 Hz, 2H), 9.32 (s, 2H), 8.78 (d, *J* = 8.3 Hz, 2H), 8.26 (d, *J* = 3.7 Hz, 2H), 7.89 (d, *J* = 8.4 Hz, 2H), 7.70 (dd, *J* = 8.0, 1.0 Hz, 2H), 7.09-7.00 (m, 2H), 6.81 (td, *J* = 7.5, 1.2 Hz, 2H), 6.28 (dd, *J* = 7.6, 1.0 Hz, 2H), 2.26 (s, 6H), 1.67 (s, 6H); ^{13}C NMR (100 MHz, Acetone-d_6) δ 184.42 , 166.53 , 154.18 , 149.89 , 142.75 , 140.60 , 134.21 , 133.43 , 130.96 , 129.21 , 128.29 , 127.99 , 124.40 , 123.26 , 113.34 , 100.89 , 27.44 , 11.12; HRMS: Calcd. For $C_{36}H_{30}IrN_6O_2$ $[M]^+$: 771.2059 Found: 771.2081; Anal. ($C_{36}H_{30}IrN_6O_2PF_6$) C, H, N: calcd 47.21, 3.30, 9.18; Found 47.33, 2.92, 9.01.

Complex **5**. Yield: 57%. ^{1}H NMR (400 MHz, Acetone-d_6) δ 8.58-8.56 (d, *J* = 8.0 Hz, 2H), 8.26 (s, 2H), 8.67 (s, 2H), 8.21-8.19 (*d*, *J* = 8.0 Hz 2H), 8.06-8.04 (d, *J* = 8.0 Hz, 2H), 7.98-7.96 (d, *J* = 8.0 Hz, 2H), 7.83-7.81 (t, *J* = 4.0 Hz, 2H), 7.63-7.60 (m, 10H), 7.16-7.14 (t, *J* = 4.0 Hz, 2H), 7.10-6.98 (d, *J* = 8.0 Hz, 2H), 6.97-6.95 (t, *J* = 4.0Hz,

2H), 6.71-6.99 (d, J = 8.0Hz, 2H), 2.07 (s, 6H); ^{13}C NMR (100 MHz, Acetone-d_6) δ 169.6, 162.8, 151.4, 150.5, 149.4, 148.7, 146.7, 140.0, 136.6, 134.1, 130.8, 130.7, 130.5, 130.0, 127.8, 127.0, 126.4, 124.8, 123.9, 118.7, 26.3; HRMS: Calcd. for $C_{48}H_{36}IrN_4$ [M–PF$_6$]$^+$: 861.2569, Found: 861.2553; Anal. ($C_{48}H_{36}N_4IrPF_6$ + H_2O) C, H, N: calcd. 56.30, 3.74, 5.47; found 56.04, 3.42, 5.49.

Complex **6**. Reported[5]

Complex **7**. Reported[6]

Complex **8**. Yield: 56%. ^{1}H NMR (400 MHz, CD_3CN-d_3) δ 8.81-8.79 (d, J = 8.0 Hz, 1H), 8.68-8.67 (d, J = 4.0 Hz, 1H), 8.61-8.60 (d, J = 4.0 Hz, 1H), 8.48-8.46 (d, J = 8.0 Hz, 1H), 8.41-8.34 (m, 4H), 8.25-8.22 (d, J = 8.0 Hz, 2H), 8.15 (s, 1H), 7.98-7.95 (q, J = 4.0 Hz, 1H), 7.88-7.85 (q, J = 4.0 Hz, 1H), 7.75-7.73 (d, J = 8.0 Hz, 2H), 7.28-7.24 (m, 4H), 7.21-7.17 (t, J = 8.0 Hz, 2H), 6.92-6.87 (t, J = 8.0 Hz, 2H), 6.86-6.81 (t, J = 8.0 Hz, 2H), 6.69-6.66 (d, J = 8.0 Hz, 2H); ^{13}C NMR (100 MHz, CD_3CN-d_3) δ 171.2, 150.7, 150.2, 148.5, 147.1, 147.0, 141.24, 141.21, 139.0, 136.5, 135.7, 135.6, 132.1, 131.7, 131.6, 131.5, 131.0, 130.1, 129.7, 128.6, 128.58, 128.4, 128.39, 128.1, 127.6, 125.1, 125.0, 124.0, 119.0; HRMS: Calcd. for $C_{42}H_{27}IrN_4Cl$ [M–PF$_6$]$^+$: 815.1542 Found: 815.1535; Anal. ($C_{42}H_{27}IrN_4ClPF_6$+H_2O) C, H, N: calcd 51.56, 2.99, 5.73; Found 51.75, 2.92, 5.90.

Complex **9**. Reported[7]

Complex **10**. Yield: 59%. ^{1}H NMR (400 MHz, Acetone-d_6) δ 8.73 (d, J = 5.6 Hz, 2H), 8.54 (d, J = 8.4 Hz, 2H), 8.48 (d, J = 8.4 Hz, 2H), 8.41 (s, 2H), 8.30 (d, J = 1.2 Hz, 2H), 8.27 (d, J = 8.0 Hz, 2H), 7.81 (d, J = 8.0 Hz, 2H), 7.33-7.27 (m, 4H), 7.22 (t, J = 8.0 Hz, 2H), 6.97 (t, J = 7.6 Hz, 2H), 6.88 (t, J = 9.8 Hz, 2H), 6.65 (d, J = 7.2 Hz, 2H); ^{13}C NMR (100 MHz, Acetone-d_6) δ 171.1, 150.9, 150.1, 148.4, 148.3, 147.0, 145.8, 141.3, 135.6, 131.9, 131.5, 130.1, 130.0, 128.8, 128.7, 128.4, 127.7, 126.0, 125.0, 124.1, 119.0; HRMS: Calcd. for $C_{42}H_{26}Cl_2IrN_4$[M–PF$_6$]$^+$: 849.1164, Found: 849.1168.

Anal.: ($C_{42}H_{26}Cl_2IrN_4PF_6 + H_2O$) C, H, N: calcd. 49.81, 2.79, 5.53; found 49.63, 2.85, 5.47.

Complex **11**. Yield: 58%. ^{1}H NMR (400 MHz, Acetone-d_6) δ 8.53 (d, J = 8.4 Hz, 2H), 8.47 (d, J = 8.8 Hz, 2H), 8.35 (d, J = 8.8 Hz, 2H), 8.06 (d, J = 8.0 Hz, 2H), 7.88 (d, J = 8.0 Hz, 2H), 7.82-7.79 (m, 4H), 7.44 (d, J = 8.8 Hz, 2H), 7.37 (t, J = 1.2 Hz, 2H), 7.08 (t, J = 8.0 Hz, 2H), 6.99 (t, J = 8.0 Hz, 2H), 6.81 (t, J = 1.2 Hz, 2H), 6.49 (d, J = 8.0 Hz, 2H), 2.81 (s, 6H); ^{13}C NMR (100 MHz, Acetone-d_6) δ 171.8, 165.4, 149.2, 148.9, 148.6, 147.1, 141.0, 139.5, 134.0, 131.5, 131.1, 130.1, 130.0, 128.6, 128.4, 128.0, 127.4, 127.3, 124.8, 123.5, 118.2, 25.2; HRMS: calcd. for $C_{44}H_{32}IrN_4[M–PF_6]^+$: 809.2256 found: 809.2304. Anal.: ($C_{44}H_{32}IrN_4PF_6$+$2H_2O$) C, H, N: calcd. 53.38, 3.67, 5.66; found 53.10, 3.50, 5.65.

Complex **12**. Yield: 63%. ^{1}HNMR (400 MHz; Acetone-d_6): δ 8.52 (d, J = 8.5 Hz, 2H), 8.34 (d, J = 8.9 Hz, 2H), 8.06 (dd, J = 7.9, 1.2 Hz, 2H), 7.96 (dd, J = 8.1, 1.4 Hz, 2H), 7.77 (s, 2H), 7.68-7.62 (m, 10H), 7.51 (dd, J = 7.4, 2.1 Hz, 4H), 7.46 (ddd, J = 8.0, 7.0, 1.0 Hz, 2H), 7.15-7.06 (m, 4H), 6.85-6.81 (m, 2H), 6.58 (dd, J = 7.8, 0.9 Hz, 2H), 2.08 (s, 6H). ^{13}C NMR (100 MHz; Acetone-d_6): δ 170.9, 163.9, 150.5, 148.75, 148.55, 147.6, 146.1, 140.0, 135.8, 133.4, 130.6, 130.1, 129.60, 129.53, 129.19, 129.10, 127.8, 127.35, 127.22, 126.8, 126.6, 124.2, 124.0, 122.6, 117.4, 24.3. MALDI-TOF-HRMS: Calcd: 961.2880, Found: 961.2846. Anal. Calcd for $C_{56}H_{40}F_6IrN_4P + 2H_2O$, C, 58.89; H, 3.88, N, 4.91, Found: C, 59.115; H, 3.58; N, 4.935.

Complex **13**. ^{1}H NMR (400 MHz, Acetone-d_6) δ 9.62 (d, J = 8.0 Hz, 2H), 9.19 (s, 2H), 8.87 (d, J = 5.2 Hz, 2H), 8.57 (d, J = 8.4 Hz, 2H), 8.49 (d, J = 8.4 Hz, 2H), 8.34 (d, J = 1.2 Hz, 2H), 8.32-8.23 (m, 2H), 7.79 (d, J = 8.2 Hz, 2H), 7.38 (d, J = 8.4 Hz, 2H), 7.27-7.23 (m, 4H), 6.92-6.71 (m, 4H), 6.69 (d, J = 0.8 Hz, 2H); ^{13}C NMR (100 MHz, Acetone-d_6) δ 171.3, 151.3, 151.1, 149.0, 148.5, 147.8, 147.1, 141.3, 140.0, 136.1, 135.6, 131.7, 131.5, 130.6, 130.1, 128.9, 128.7, 128.4, 127.6, 125.3, 124.1, 119.0; HRMS: Calcd. for $C_{44}H_{28}IrN_6[M–PF_6]^+$: 833.2005, Found: 833.1926. Anal.: ($C_{44}H_{28}IrN_6PF_6 + 2.5H_2O$) C, H, N: calcd.51.66, 3.25, 8.32; found 51.77, 3.08, 8.64.

Complex **14**. Yield: 60%. ^{1}H NMR (400 MHz, Acetone-d_6) δ 8.94 (d, J = 1.6 Hz, 2H), 8.55-8.53 (m, 4H), 8.87 (d, J = 5.2 Hz, 2H), 8.39 (d, J = 6.0 Hz, 2H), 8.28 (d, J = 7.6 Hz, 2H), 8.02 (d, J = 5.6 Hz, 2H), 7.90-7.88 (m, 6H), 7.58-7.53 (m, 8H), 7.43 (t, J = 8.0 Hz, 2H), 7.20-7.18 (m, 4H), 6.86 (t, J = 8.0 Hz, 2H), 6.61 (d, J =.8.0 Hz, 2H); ^{13}C NMR (100 MHz, Acetone-d_6) δ 171.3, 157.2, 152.3, 151.8, 149.1, 148.5, 147.0, 141.3, 136.2, 135.4, 132.0, 131.6, 131.5, 130.3, 128.9, 128.4, 127.7, 126.4, 125.8, 123.8, 122.7, 119.0; HRMS: Calcd. for $C_{52}H_{36}IrN_6[M–PF_6]^+$: 909.2569 Found: 909.2590. Anal.: ($C_{52}H_{36}IrN_6PF_6$) C, H, N: calcd.59.25, 3.44, 5.32; found 59.03, 3.63, 5.10.

Complex **15**. Yield: 54%. ^{1}H NMR (400 MHz, Acetone-d_6) δ 9.09 (d, J = 8.4 Hz, 1H), 8.89 (d, J = 7.6 Hz, 1H), 8.81 (d, J = 4.8 Hz, 1H), 8.72 (d, J = 4.8 Hz, 1H), 8.61 (s, 1H), 8.27-8.23 (m, 1H), 8.16-8.13 (m, 1H), 7.93 (d, J = 7.2 Hz, 2H), 7.55 (d, J = 8.0 Hz, 2H), 7.16 (t, J = 8.0 Hz, 2H), 7.09 (t, J = 7.6 Hz, 2H), 6.93-6.90 (m, 2H), 6.79-6.75 (m, 2H), 6.54-6.51 (m, 2H), 5.54 (t, J = 7.6 Hz, 2H); ^{13}C NMR (100 MHz, Acetone-d_6) δ 164.7, 153.1, 152.5, 149.5, 149.2, 149.0, 147.5, 139.7, 139.6, 137.9, 135.4, 134.3, 133.4, 133.3, 133.2, 131.5, 130.7, 130.6, 130.3, 129.1, 127.5, 127.4, 124.2, 124.1, 123.7, 123.6, 123.5, 122.5, 113.4, 113.3, 112.8; HRMS: Calcd. for $C_{38}H_{25}ClIrN_6[M–PF_6]^+$: 793.1458 Found: 793.1422. Anal.: ($C_{38}H_{25}ClIrN_6PF_6 + H_2O$) C, H, N: calcd.47.73, 2.85, 8.79; found 47.75, 3.16, 8.50.

Complex **16**. Yield: 53%. ^{1}H NMR (400 MHz, Acetone-d_6) δ 9.04 (dd, J = 8.5, 1.3 Hz, 1H), 8.84 (dd, J = 8.3, 1.3 Hz, 1H), 8.62 (s, 1H), 8.54 (dd, J = 5.1, 1.3 Hz, 1H), 8.46 (dd, J = 5.0, 1.4 Hz, 1H), 8.18 (dd, J = 8.5, 5.1 Hz, 1H), 8.07 (dd, J = 8.3, 5.1 Hz, 1H), 7.45-7.41 (m, 2H), 7.29 (dd, J = 7.3, 2.5 Hz, 2H), 7.20-7.13 (m, 2H), 7.06 (tt, J = 7.4, 1.2 Hz, 2H), 5.00 (dtd, J = 10.6, 8.6, 2.3 Hz, 2H), 4.60 (dtd, J = 10.6, 8.6, 5.2 Hz, 2H), 3.76 (dddd, J = 12.0, 10.7, 8.5, 3.4 Hz, 2H), 3.08 (dddd, J = 11.9, 10.8, 7.9, 3.9 Hz, 2H); ^{13}C NMR (100 MHz, Acetone-d_6) δ 181.34 , 153.95 , 153.43 , 150.56 , 150.30 , 149.60 , 148.13 , 138.31 , 135.79 , 133.87 , 133.31 , 132.18 , 131.66 , 131.19 , 130.05 , 128.21 , 128.08 , 127.63 , 122.88 , 72.39 , 50.31; MALDI-TOF-HRMS: Calcd. For $C_{30}H_{23}ClIrN_4O_2$ $[M]^+$: 699.1126, Found: 699.1136; Anal.: ($C_{30}H_{23}ClIrN_4O_2PF_6$) C, H, N: calcd.42.68, 2.75, 6.64, found 42.98, 2.87, 6.71.

Complex **17**. Reported[8]

Total cell extract preparation

The TRAMPC1 (ATCC® CRL2730™) cell line were purchased from American Type Culture Collection (Manassas, VA 20108 USA). Prostate cancer cells were trypsinized and resuspended in TE buffer (10 mM Tris-HCl 7.4, 1 mM EDTA). After incubation on ice for 10 min, the lysate was centrifuged and the supernatant was collected.

Luminescence response of Ir(III) complexes 1–17 towards different forms of DNA

The G-quadruplex DNA-forming sequence (PS2. M) was annealed in Tris-HCl buffer (20 mM Tris, 100 mM KCl, pH 7.0) and were stored at –20 °C before use. Complex **1**–**17** (1 μM) was added to 5 μM of ssDNA, dsDNA or PS2. M G-quadruplex DNA in Tris-HCl buffer (20 mM Tris, pH 7.0).

Detection of enzymes activities

The random-coil oligonucleotides ON1 (100 μM) and ON2 (100 μM) were incubated in Tris buffer (20 mM, pH 7.0). The solution was heated to 95 °C for 10 min, cooled to room temperature at 0.1 °C/s, and further incubated at room temperature for 1 h to ensure formation of the duplex substrate. The annealed product was stored at –20 °C before use. For assaying enzyme activity, 50 μL of Tris buffered solution (5 mM Tris-HCl, 5 mM NaCl, 1 mM $MgCl_2$, 1 mM ATP, 0.1 mM DTT, pH 7.9) with the indicated concentrations of helicase or S1, Endo, DpnI, ExoI, EcoRI, RNase, DNase, and SSB were added to a solution containing the duplex substrate (0.25 μM). The mixture was heated to 37 °C for 2 h to allow the indicated enzymes-catalyzed unwinding of the duplex substrate to take place. The duplex unwinding reaction was quenched by the addition of EDTA at a final concentration of 20 mM, and the mixture was subsequently diluted using Tris buffer (20 mM Tris, 20 mM KCl, 150 mM NH_4Ac, pH 7.2) to a final volume of 500 μL. Finally, 1 μM of complex **9** or suramin,

TBBT and ciprofloxacin were added to the mixture. Emission spectra were recorded in the 500−720 nm range using an excitation wavelength of 360 nm.

For the detection of helicase activity in cell extract, 50 μL of Tris buffered solution (5 mM Tris-HCl, 5 mM NaCl, 1 mM $MgCl_2$, 1 mM ATP, 0.1 mM DTT, pH 7.9) and the indicated concentrations of helicase were added to a solution containing the duplex substrate (0.25 μM) and cell extract. The mixture was heated to 37 °C for 2 h to allow the helicase-catalyzed unwinding of the duplex substrate to take place. The duplex unwinding reaction was quenched by the addition of EDTA at a final concentration of 20 mM, and the mixture was subsequently diluted using Tris buffer (20 mM Tris, 20 mM KCl, 150 mM NH_4Ac, pH 7.2) to a final volume of 500 μL. Finally, 1 μM of complex **9** was added to the mixture. Emission spectra were recorded in the 500−720 nm range using an excitation wavelength of 360 nm.

Table S1. DNA sequences used in this project:

	Sequence
ON1	5′- $GTG_3TAG_3CG_3T_2G_2TG_2CGA$<br>$CG_2CAGCGAG_2CAGAG_2AGCAGAG_3AGCA$-3′
ON2	5′- GC_2TCG $CTGC_2GTCGC_2AC_2A_2C_3GC_3$-3′
PS2.M	5′-GTGGGTAGGGCGGGTTGG-3′
CCR5-DEL	5′-$CTCAT_4C_2ATACAT_2A_3GATAGTCAT$-3′
ds17	5′-$C_2AGT_2CGTAGTA_2C_3$-3′
	5′-$G_3T_2ACTACGA_2CTG_2$-3′
F21T	5′-FAM-($G_3[T_2AG_3]_3$)-TAMRA-3′
F10T	5′-FAM-TATAGCTA-HEG-TATAGCTATAT-TAMRA-3′
$ON1_m$	5′- $GT\underline{\mathit{ATA}}TA\underline{\mathit{TAC}}CG_3T_2G_2TG_2CGACG_2CAGCGAG_2C$<br>$AGAG_2AGCAGAG_3AGCA$-3′

Table S2 Photophysical properties of iridium(III) complexes **1**–**17**.

Complex	Quantum yield	λ_{em}/ nm	Lifetime/ μs	UV/vis absorption λ_{abs} / nm (ε/ dm^3 mol^{-1} cm^{-1})
**1**	0.13	629	3.29	269 (4.19 × 10^4), 354 (1.27 × 10^4), 370 (1.54 × 10^5)
**2**	0.057	577	0.74	261 (3.3 × 10^4), 268 (3.2 × 10^3), 296 (1.9 × 10^4), 371 (9.05 × 10^3)
**3**	0.089	567	4.16	261 (1.24 × 10^4), 311 (5.48 × 10^3), 348 (1.73 × 10^3)
**4**	0.015	578	1.53	231 (3.49 × 10^4), 270 (2.56 × 10^4), 339 (5.32 × 10^3)
**5**	0.12	590	1.23	336 (1.42 × 10^4)
**6**	0.089	580	0.28	236 (1.56 × 10^5), 285 (7.75 × 10^4), 302 (6.43 × 10^4)
**7**	0.056	571	1.34	278 (1.33 × 10^4), 332 (4.67 × 10^3)
**8**	0.27	583	4.31	280 (3.6 × 10^4), 429 (5.9 × 10^3)
**9**	0.12	570	8.13	270 (5.72 × 10^4), 333 (2.06 × 10^4),
**10**	0.086	590	2.89	263(2.90 × 10^4), 278 (2.99 × 10^4), 332 (1.10 × 10^4)
**11**	0.087	570	1.96	270 (3.13 × 10^4), 337 (2.33 × 10^4)
**12**	0.063	575	1.84	234 (2.55 × 10^4), 262 (2.20 × 10^4), 286 (2.67 × 10^4), 350 (7.91 × 10^3)
**13**	0.067	568	4.61	262 (3.79 × 10^4), 279 (2.88 × 10^4), 334 (1.13 × 10^4)
**14**	0.15	560	4.586	278 (1.34 × 10^5), 355 (1.92 × 10^4), 454 (4.0 × 10^3)
**15**	0.092	620	2.71	274 (7.38 × 10^3), 301 (6.31 × 10^3), 372 (1.93 × 10^3)
**16**	0.069	588	1.09	230 (2.73 × 10^4), 270 (1.64 × 10^4), 345 (3.33 × 10^3)
**17**	0.078	608	2.87	235 (1.69 × 10^4), 252 (1.81 × 10^4), 266 (1.94 × 10^4)

Fig. S1 Diagrammatic bar array representation of the luminescence enhancement selectivity ratio of complexes **1**–**7** for PS2. M G-quadruplex DNA over dsDNA (ds17) and ssDNA (CCR5-DEL).

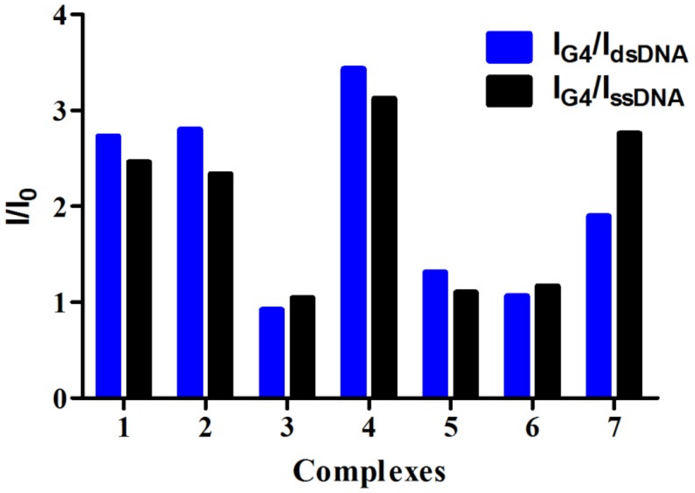

Fig. S2 Diagrammatic bar array representation of the luminescence enhancement selectivity ratio of complexes **7**–**17** for PS2. M G-quadruplex DNA over dsDNA (ds17) and ssDNA (CCR5-DEL).

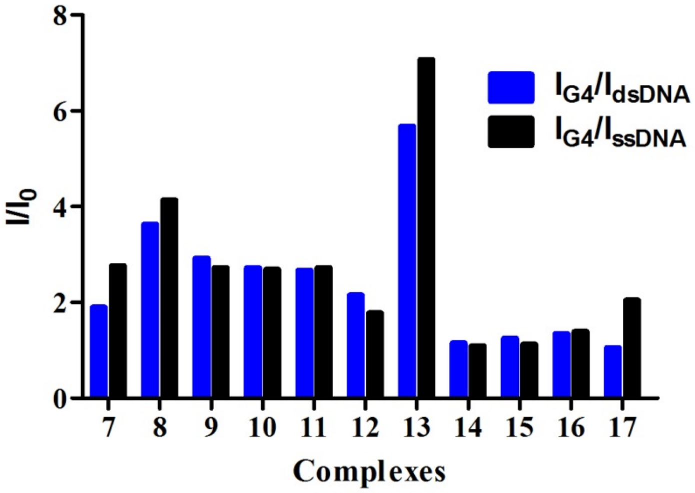

Fig. S3 (a) Melting profile of F21T G-quadruplex DNA (0.2 μM) in the absence and presence of **9** (5 μM). (b) Melting profile of F10T dsDNA (0.2 μM) in the absence and presence of **9** (5 μM).

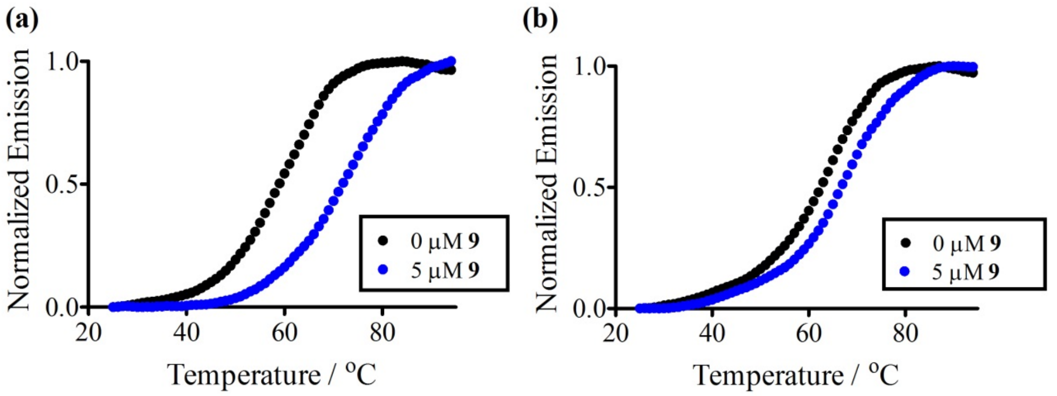

Fig. S4 Emission spectrum of the system with complex **9** alone ([complex **9**] = 1 μM) in the absence and presence of helicase (0.9 μM).

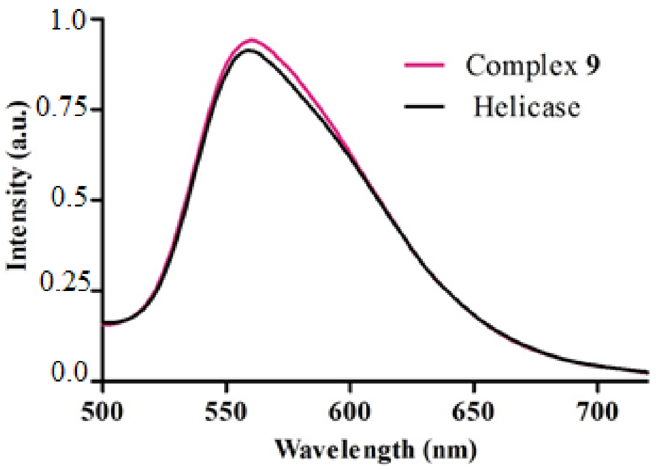

Fig. S5 Emission spectrum of complex **9** (1 μM) in the presence of helicase (0.9 μM) and $ON1_m$/ON2 duplex mutant (0.25 μM).

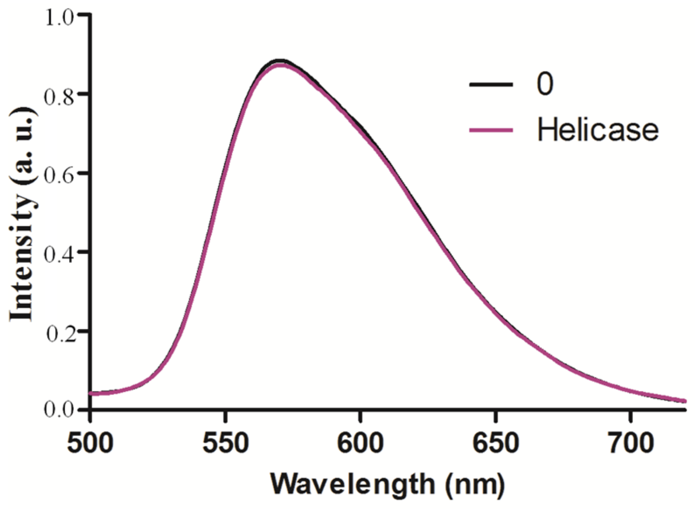

Fig. S6 Relative luminescence response of **9**/G-quadruplex ensemble upon the addition of 0.8 μM HCV NS3 helicase.

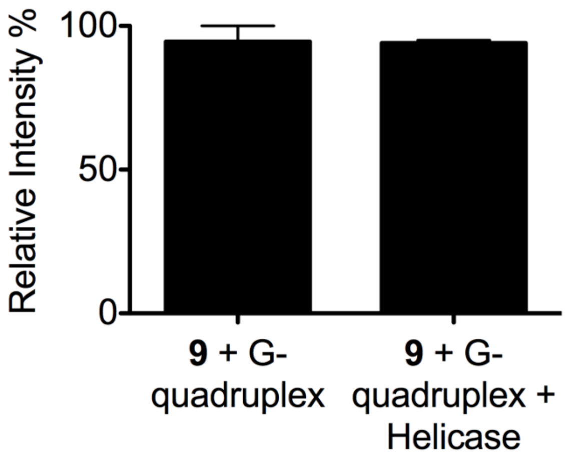

Fig. S7 Relative luminescence response of the system in the absence or presence of helicase (0.9 μM) at various concentrations of complex **9** (0.25, 0.5, 1 and 2 μM). 1 μM of complex **9** offered the highest luminescence fold-change response compared to 0.25, 0.5 or 2 μM of complex **9**.

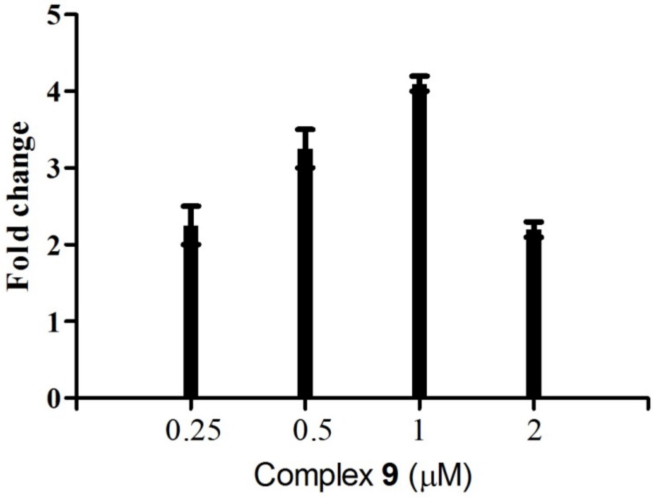

Fig. S8 Relative luminescence response of the system in the absence or presence of helicase (0.9 μM) at various concentrations of duplex DNA (0.125, 0.25, 0.5, and 1 μM). It was observed that the luminescence response of the system was highest at 0.25 μM of duplex DNA.

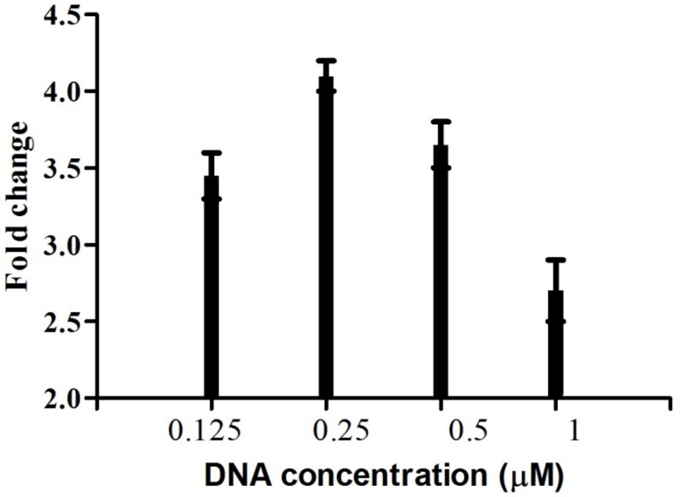

Fig. S9 Relative luminescence response of the system in the absence or presence of helicase (0.9 μM) at various concentrations of ATP (0.2, 0.5, 1, and 2.5 mM). It was observed that the luminescence response of the system was highest at 1 mM of ATP.

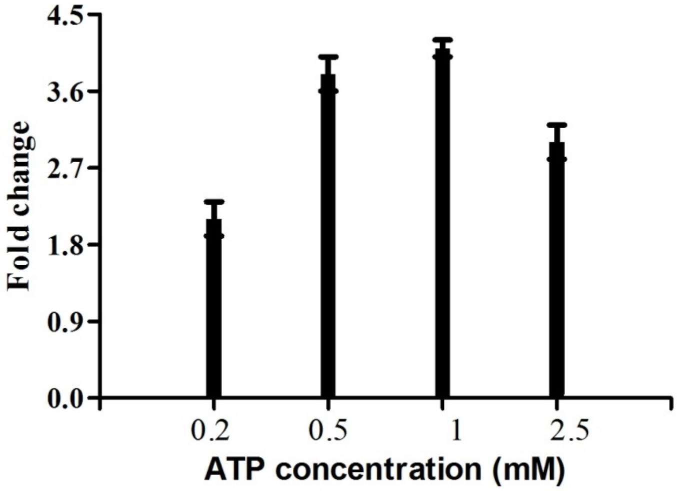

Fig. S10 Emission spectral traces of complex **9** (1 μM) and duplex DNA (0.25 μM) upon incubation with helicase (0.09 μM) in Tris-HCl buffer (20 mM, 50 mM KCl, 150 mM NH_4Ac, pH 7.2), showing a signal-to-noise ratio greater than 3.

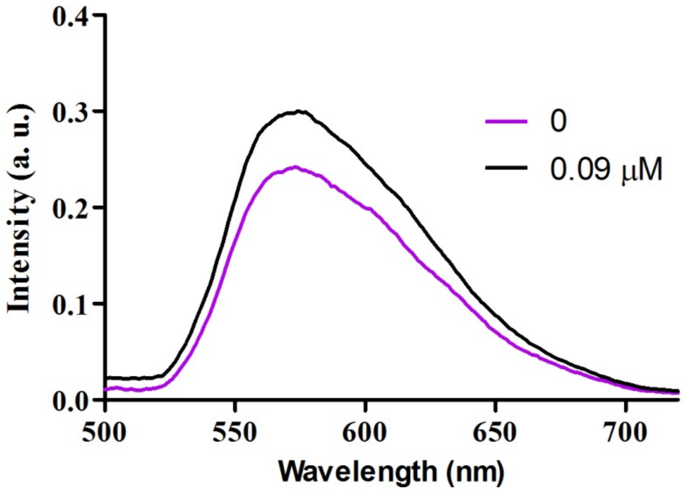

Fig. S11 (a) Relative luminescence response of complex **9** in the absence and presence of 10 μM of suramin and TBBT. (b) Relative luminescence response of the **9**/G-quadruplex ensemble upon the addition of 10 μM of suramin and TBBT.

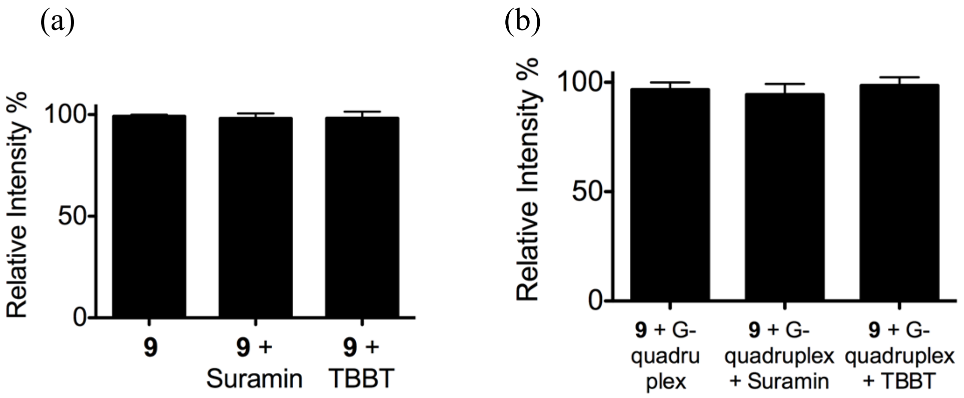

References

1. G. A. Crosby and J. N. Demas, *J. Phys. Chem.*, 1971, **75**, 991-1024.
2. D. Monchaud, C. Allain, H. Bertrand, N. Smargiasso, F. Rosu, V. Gabelica, A. De Cian, J. L. Mergny and M. P. Teulade-Fichou, *Biochimie*, 2008, **90**, 1207-1223.
3. Q. Zhao, S. Liu, M. Shi, C. Wang, M. Yu, L. Li, F. Li, T. Yi and C. Huang, *Inorg. Chem.*, 2006, **45**, 6152-6160.
4. D.-L. Ma, L.-J. Liu, K.-H. Leung, Y.-T. Chen, H.-J. Zhong, D. S.-H. Chan, H.-M. D. Wang and C.-H. Leung, *Angew. Chem. Int. Ed.*, 2014, DOI:10.1002/anie.201404686.
5. F. Gärtner, S. Denurra, S. Losse, A. Neubauer, A. Boddien, A. Gopinathan, A. Spannenberg, H. Junge, S. Lochbrunner, M. Blug, S. Hoch, J. Busse, S. Gladiali and M. Beller, *Chem. Eur. J.*, 2012, **18**, 3220-3225.
6. K.-H. Leung, H.-Z. He, V. P.-Y. Ma, D. S.-H. Chan, C.-H. Leung and D.-L. Ma, *Chem.Comm.*, 2013, **49**, 771-773.
7. C. Dragonetti, L. Falciola, P. Mussini, S. Righetto, D. Roberto, R. Ugo, A. Valore, F. De Angelis, S. Fantacci, A. Sgamellotti, M. Ramon and M. Muccini, *Inorg. Chem.*, 2007, **46**, 8533-8547.
8. P. M. Griffiths, F. Loiseau, F. Puntoriero, S. Serroni and S. Campagna, *Chem.Comm.*, 2000, 2297-2298.
